# Comparison of Mortality Risk With Different Surgeon and Hospital Operative Volumes Among Individuals Undergoing Pancreatectomy by Emulating Target Trials in US Medicare Beneficiaries

**DOI:** 10.1001/jamanetworkopen.2022.1766

**Published:** 2022-03-10

**Authors:** Arin L. Madenci, Kerollos Nashat Wanis, Zara Cooper, S. V. Subramanian, Sebastien Haneuse, Albert Hofman, Miguel Hernán

**Affiliations:** 1 Department of Epidemiology, Harvard T.H. Chan School of Public Health, Boston, Massachusetts; 2 Department of Surgery, Brigham and Women’s Hospital, Boston, Massachusetts; 3 CAUSALab, Harvard T.H. Chan School of Public Health, Boston, Massachusetts; 4 Department of Social and Behavioral Sciences, Harvard T.H. Chan School of Public Health, Boston, Massachusetts; 5 Department of Biostatistics, Harvard T.H. Chan School of Public Health, Boston, Massachusetts

## Abstract

**Importance:**

The association of surgeons’ and hospitals’ operative volumes with postoperative patient outcomes has been studied for decades and holds important policy implications; however, in many volume-outcome analyses, this association is described without the envisioning of a clear intervention, which often introduces unintentional bias. Acting on such results may lead to unintended consequences from policy interventions or patient recommendations.

**Objective:**

To specify how (hypothetical) target trials would be designed to estimate the association between postoperative mortality of patients undergoing operations and a range of surgeon and hospital volume conditions and then to emulate these trials by using observational data.

**Design, Setting, and Participants:**

This observational data analysis emulated 4 hypothetical target trials of increasing complexity, ranging from a poorly defined trial that would randomly assign participants only to surgeon volume to one that would randomly assign participants to surgeon volume, hospital volume, and specific surgeon and hospital. This population-based cohort study included 9136 Medicare beneficiaries with a first diagnosis of pancreatic malignant neoplasm who did not require neoadjuvant therapy and underwent pancreatectomy between January 1, 2012, and September 30, 2016. Data analysis was performed between September 1, 2019, and October 8, 2021.

**Exposures:**

Number of pancreatectomies performed by surgeon and hospital during the prior year.

**Main Outcomes and Measures:**

Ninety-day mortality.

**Results:**

The analyses included 9136 Medicare beneficiaries treated by 1358 surgeons at 697 hospitals; median age was 73.3 years (IQR, 69.1-78.1 years), and 4642 were men (51%). When trials with poorly defined interventions on surgeon volume were emulated, the estimated 90-day mortality was 7.9% (95% CI, 6.4%-9.4%) for lower-volume surgeons and 5.2% (95% CI, 2.7%-10.9%) for higher-volume surgeons. When trials with better-defined interventions were emulated, the difference was reduced: 7.8% (95% CI, 6.3%-9.3%) for lower-volume surgeons and 7.2% (95% CI, 6.0%-8.7%) for higher-volume surgeons.

**Conclusions and Relevance:**

In this cohort study that emulated 4 different target trials with data from Medicare beneficiaries undergoing pancreatectomy, mortality differences across surgical volume levels were attenuated when the interventions were well defined. The application of the hypothetical target trial framework to this specific volume-outcomes scenario revealed the complexities of this research question and the unintentional biases introduced in prior studies, which emulated poorly defined trials whose results are therefore difficult to interpret. The target trial framework may be of value to outcomes researchers asking questions that correspond to well-defined interventions for the real world.

## Key Points

**Question:**

How can past studies of the volume-outcomes association in surgery be interpreted and how might future research on these questions generate valid results?

**Findings:**

In this cohort study that emulated 4 hypothetical target trials among 9136 Medicare beneficiaries undergoing pancreatectomy for malignancy, mortality was higher for higher volumes only when target trials with poorly defined interventions were emulated.

**Meaning:**

The target trial framework demonstrated in this study may be useful for volume-outcomes researchers who are not willing to make unrealistic assumptions in their studies.

## Teaser

This cohort study analyzes 4 hypothetical target trials of Medicare beneficiaries with pancreatic malignant neoplasms to analyze postoperative mortality differences across surgical and hospital operative volumes.

## Introduction

Efforts to make surgery safer often focus on operative volume, or the frequency with which a surgeon or hospital has performed a certain operation. Previous studies have suggested that higher hospital and surgeon volumes have a beneficial association with postoperative outcomes. A correlation between the “number of surgical procedures done in a hospital and the in-hospital mortality rate for those patients” was first reported in 1979.^1^^(p1367)^ Subsequently, other studies^2,3,4^ echoed these findings, including a large-scale study of Medicare beneficiaries concluding that “patients can often improve their chances of survival substantially, even at high-volume hospitals, by selecting surgeons who perform the operations frequently.”^5^^(p2117)^

In response to these reports, patient safety organizations, such as the Leapfrog Group, recommended minimum hospital and surgeon volume standards for credentialing certain procedures,^6,7^ which have since been adopted by several major academic hospital networks.^8^ However, the findings that support these rules are based on simplistic and relatively unrealistic comparisons of high- vs low-volume surgeons and institutions.

Among patients planning to undergo elective resection of a pancreatic malignant neoplasm, we specified 4 hypothetical randomized trials—target trials—with increasingly better-defined interventions on operative volumes. Each of these trials was then emulated with observational data from Medicare claims. In sequentially considering these 4 scenarios, we demonstrated how the hypothetical target trial framework may be of value to outcomes researchers asking research questions that correspond to well-defined interventions for the real world.

## Methods

### Specification of the Target Trials

A randomized trial to estimate the effect of operative volume may never be implemented because of logistic challenges (eg, coordination of patient and surgeon schedules), enrollment difficulties (eg, patients may not want to be assigned to an unfamiliar surgeon or hospital), financial problems (eg, funding for travel and lodging for patients who must seek care far from home), and other challenges. Because of these impediments in conducting a randomized trial, observational data analyses are the best chance to inform decisions about surgeon and hospital volume.

A helpful tool to strengthen the validity of observational analyses is to specify the hypothetical randomized trial that one would like to conduct—the target trial^9^—and then try to emulate it by using observational data. This section outlines the design of 4 hypothetical target trials in which patients with a first diagnosis of pancreatic malignant neoplasm are randomly assigned to select a surgeon with a particular operative volume in the past year.

For each of the hypothetical trials, patients (this article refers to trial participants as “patients” instead of “individuals” to avoid confusion with an intervention on surgeons, which would require a separate analysis) will be eligible if they are older than 65 years, do not have evidence of metastatic disease, and are not expected to receive chemotherapy preoperatively. All patients will be followed up for 3 months or until death, whichever occurs earlier, and the risk of all-cause mortality will be compared between groups.

Each of the 4 trials will randomly assign trial participants to selecting a surgeon who performed a particular number of operations in the past year. The method to select the surgeon will vary across trials, as described later. For all trials, we assume that we will have knowledge of the number of pancreatic resections for malignant neoplasms that each surgeon in the United States performed during the previous year. Similar information has been made available by organizations such as ProPublica^10^ and the New York Department of Health.^11^

If these hypothetical trials were conducted, intention-to-treat estimates of 90-day mortality risk could be calculated nonparametrically or with standard logistic models (eMethods in the Supplement). Alternatively, nonnaive per-protocol estimates, or the mortality risk that would have been observed had all participants been adherent to their assigned strategy, could be estimated by adjusting for prognostic covariates also associated with adherence.

#### Target Trial 1: Assignment to Surgeon Volume

Eligible patients are randomly assigned to a specific surgeon volume ranging from 0 to 25 pancreatic resections per year; that is, the trial has 26 arms. Patients must select their surgeon from available surgeons with their assigned volume (based on, for example, proximity, reputation, or years in practice), but must be ready to travel, sometimes long distances, to reach the chosen surgeon. The directed acyclic graph in eFigure 1A in the Supplement represents key components of this trial.

This trial would be of little interest to policy makers and patients because the interventions do not consider the hospital context in which surgeons operate. If, for example, surgeons’ operative volumes were associated with the operative volume of the hospital where they work and hospital volume had a direct effect on the outcome (eg, because hospitals with greater volumes have better-trained multidisciplinary teams, regardless of a particular surgeon’s experience), this trial would not provide useful information for decision-making. A more useful trial would take into account the hospital volume in addition to the surgeon volume.

#### Target Trial 2: Assignment to Surgeon Volume and Hospital Volume

Eligible patients are randomly assigned to a surgeon volume ranging from 0 to 25 pancreatic resections per year and to a hospital volume ranging from 0 to 45 pancreatic resections per year; that is, the trial has 1196 arms. This trial is represented by the directed acyclic graph in eFigure 1B in the Supplement.

Although this trial defines the interventions by both surgeon volume and hospital volume, patients may still be required to travel, sometimes long distances, to reach a surgeon and hospital fulfilling their assignment criteria. Therefore, implementing this trial would be impractical. A more useful trial would take into account the distance between a patient and the surgeon and hospital with the requisite operative volumes.

#### Target Trial 3: Assignment to Nearby Surgeon Volume and Hospital Volume

Eligible patients are randomly assigned to 1 of the strategies specified in trial 2, but if the nearest available surgeon and hospital are at least 1.5 hours’ driving distance away, then the patient is assigned to undergo an operation by the surgeon and hospital with the volumes closest to those assigned within 1.5 hours’ driving distance. If no surgeon or hospital is available within 1.5 hours, patients may select their surgeon and hospital by criteria of their choosing. This trial is represented by the directed acyclic graph in eFigure 1C in the Supplement.

This trial is better defined than the previous ones, but its results would be hard to interpret if there existed certain savvy patients who knew how to select the best available surgeon or hospital (among those available to them under their assigned strategy) and if these patients were also healthier. A noncausal association between the specific surgeon (or hospital) selected by a patient and that patient’s probability of mortality would follow.

A more useful trial would prevent patients from using their knowledge about characteristics of surgeons or hospitals to select them. Otherwise, the trial estimates would not be transportable to a future population (or another country’s population) with a different distribution of surgeon or hospital characteristics.

#### Target Trial 4: Double Assignment to Nearby Surgeon and Hospital Volume and to a Particular Surgeon and Hospital

Eligible patients are first randomly assigned to one of the strategies specified in trial 3, and then randomly assigned to a particular surgeon and hospital among available ones. In this way, the effect of assignment to a specific operative volume of a surgeon and hospital is decoupled from the distribution of surgeon and hospital characteristics. (For another example of sequentially randomizing, in the setting of organ transplantation, see Wanis and colleagues.^9^) A directed acyclic graph for trial 4 is shown in eFigure 1D in the Supplement.

### Emulation of the Target Trials

The aforementioned protocols may never be conducted as randomized trials because of the difficulty of enrolling many thousands of patients willing to adhere to an intervention potentially requiring them to trust a surgeon they have never met from an unfamiliar hospital far from home. However, researchers can attempt to emulate these trials by using observational data from Medicare, a federal health insurance program that provides coverage for approximately 96% of US citizens aged 65 years or older. This section describes how to emulate key aspects of the target trials; numeric results are presented in the next section. This study was approved by the Harvard T.H. Chan School of Public Health institutional review board, and its reporting follows the Strengthening the Reporting of Observational Studies in Epidemiology (STROBE) reporting guideline. Participant race and ethnicity were derived from the Medicare claims database. Patient consent was not required or obtained for this database study. Because the study met criteria for exemption, the need for informed consent was waived by the institutional review board.

#### Eligibility Criteria and Treatment Strategies

The eligibility criteria are the same as those described for the target trials. The treatment strategies in the emulations were the same as described earlier, with an important modification. In a truly randomized trial, after being selected, a surgeon may deem the patient inoperable because of perioperative risk from comorbidities (eg, severe congestive heart failure), short life expectancy, or extent of malignancy (ie, surgeon judgment about whether a tumor is borderline resectable vs unresectable); or the patient may progress to unresectable disease or develop severe comorbidities in the period between surgeon selection and tumor resection. In both cases, the operation may be canceled.

#### Treatment Assignment

Eligible patients were assigned to the strategy that was consistent with their observed data. For example, for the emulation of trial 1, a patient operated on by a surgeon who conducted 10 pancreatic resections per year was assigned to the group “surgeon volume 10.” For the emulation of trials 3 and 4, patients at least a 1.5-hour drive from any surgeon were assigned to their observed surgeon and hospital volume (ie, the natural value of treatment). The proportion of patients within the travel time thresholds by surgeon and hospital volume is summarized in eFigure 2 in the Supplement. In sensitivity analyses for the emulation of trials 3 and 4, the driving time threshold of 1.5 hours was replaced by thresholds of 3 and 6 hours.

To attempt to emulate a randomized assignment of surgeon and hospital volume, the analysis adjusted for baseline covariates expected to be prognostic for postoperative mortality and associated with surgeon (and hospital) operative volume as described in the Statistical Analysis section (details of covariates are available in the eAppendix in the Supplement). For the emulation of trial 4, the analysis also attempted to emulate randomization to a specific surgeon and hospital within levels of operative volume by adjusting for characteristics of available surgeons (age, sex, and working at more than 1 hospital) and hospitals (availability of a cardiac intensive care unit, payment type, teaching category, and size).

#### Follow-up and Outcome

For each patient, follow-up started at pancreatectomy and ended 90 days later or at death, whichever occurred earlier. No patients were lost to follow-up. Death from any cause was assessed by linkage with the Medicare Vital Status File.

### Statistical Analysis

The analyses estimated the observational analog of the per-protocol effect size (equivalent to the risk difference that would be observed had all participants been adherent to their assigned strategy) from a standard logistic model (eMethods in the Supplement). Data analysis was performed between September 1, 2019, and October 8, 2021.

For the emulation of trials 1 and 2, risk was estimated with a logistic regression model with indicators for treatment group and the patient’s baseline covariates (Table 1), and then the estimated risks were standardized to the overall population. To obtain valid 95% CIs in the presence of clustering by surgeon and hospital, a nonparametric cluster bootstrap procedure with 1000 resamples was used.

**Table 1.** Characteristics of 9136 Individuals Who Underwent Elective Pancreatic Resection for Malignant Neoplasms Reimbursed by Medicare, 2012-2016

Characteristic	Patients, No. (%)
Age, median (IQR), y	73.3 (69.1-78.1)
Sex	
Men	4642 (51)
Women	4494 (49)
Race	
Black	600 (7)
White	8130 (89)
Other^a^	406 (4)
Acute myocardial infarction	303 (3)
Dementia	435 (5)
Atrial fibrillation	1197 (13)
Chronic kidney disease	2435 (27)
Chronic obstructive pulmonary disease	1918 (21)
Congestive heart failure	1710 (19)
Diabetes	4122 (45)
Coronary artery disease	4318 (47)
Stroke or transient ischemic attack	832 (9)
Inpatient at operation	576 (6)
Annual volume, median (IQR)	
Hospital	12.0 (5.0-23.0)
Surgeon	5.0 (3.0-10.0)
Year	
2012	1810 (20)
2013	1885 (21)
2014	1864 (20)
2015	1944 (21)
2016	1633 (18)

^a^ Includes Asian (134 individuals), Hispanic (63 individuals), North American Native (25 individuals), or other race and ethnicity unspecified in the Medicare database (184 individuals).

The aforementioned analyses would not have correctly emulated the trials in the likely presence of confounding by driving time. However, adjustment for driving time would result in unstable estimates because of the rarity of some combinations of volume and driving time. For example, in these data, among patients residing more than 3 hours away from a surgeon with the average operative volume (3 operations in the past year), only 3 underwent pancreatectomy by a surgeon with a volume of at least 15 operations per year. This quasi-nonpositivity is not a problem for the emulation of trial 3 because driving time is explicitly incorporated into the strategies and thus no adjustment for it is required.

To emulate the second randomized assignment in trial 4, the aforementioned procedure was modified to add further adjustment for surgeon- and hospital-level covariates (Table 2), in addition to the patient covariates that were previously included. Software code for this analysis is available on GitHub.^12^ Additional details are available in the eMethods in the Supplement.

**Table 2.** Characteristics of Surgeons and Hospitals Who Performed Elective Pancreatic Resection for Malignant Neoplasms Reimbursed by Medicare, 2013-2016

Characteristic	No. (%)
No. of hospitals	697
Annual hospital volume, mean (range)	6 (1-139)
Cardiac intensive care unit	505 (72)
Hospital type	
For profit	89 (13)
Nonprofit	534 (77)
Government nonfederal	74 (11)
Teaching category	
Major	210 (30)
Minor	278 (40)
Nonteaching	209 (30)
Size (No. of beds)	
Small (1-99)	15 (2)
Medium (100-399)	342 (49)
Large (≥400)	340 (49)
No. of surgeons	1358
Annual surgeon volume, mean (range)	3 (1-50)
Surgeon age, mean (range), y	50 (31-84)
Female surgeons	105 (8)
Operate at >1 hospital	226 (17)

## Results

We identified 9136 patients who met the aforementioned eligibility criteria between January 1, 2012, and September 30, 2016. Median age was 73.3 years (IQR, 69.1-78.1 years), 4642 were men (51%), 4494 were women (49%), 600 were Black (7%), 8130 were White (89%), and 406 (4%) were Asian (134 [1%]), Hispanic (63 [1%]), North American Native (25 [0.3%]), or other race and ethnicity unspecified in the Medicare database (184 [2%]). Their characteristics are summarized in Table 1. The characteristics of the 1358 treating surgeons and 697 treating hospitals are summarized in Table 2.

### Surgeon Operative Volume

At median-volume hospitals (or the natural value of hospital volume for trial 1), risk of 90-day mortality with higher- and lower-volume surgeons ranged from 5.2% (95% CI, 2.7%-10.9%) to 7.9% (95% CI, 6.4%-9.4%) in the emulation of trial 1, from 4.5% (95% CI, 2.1%-9.3%) to 6.8% (95% CI, 4.8%-9.0%) in the emulation of trial 2, from 7.2% (95% CI, 6.0%-8.7%) to 7.8% (95% CI, 6.3%-9.3%) in the emulation of trial 3, and from 7.1% (95% CI, 5.9%-8.6%) to 7.9% (95% CI, 6.5%-9.6%) in the emulation of trial 4. Estimates for each of the 4 target trial emulations are further summarized in Table 3 and the Figure.

**Table 3.** Estimates and Risk Differences in 90-Day Mortality by Surgeon and Hospital Volume for Each Emulated Target Trial^a^

Variable	Surgeon volume 2/y^b^	Surgeon volume 5/y	Surgeon volume 18/y	Surgeon volume 25/y
**Hospital: natural value**				
Trial 1				
Estimate, % (95% CI)	7.9 (6.4 to 9.4)	7.5 (5.9 to 9.5)	6.6 (4.4 to 10.2)	5.2 (2.7 to 10.9)
Risk difference (95% CI), percentage points	Reference	−0.3 (−2.1 to 1.9)	−1.2 (−4.5 to 3.0)	−2.6 (−5.7 to 3.2)
**Hospital: 2/y**				
Trial 2				
Estimate, % (95% CI)	11.0 (9.1 to 12.9)	10.5 (7.5 to 14.4)	9.3 (5.5 to 14.6)	7.4 (3.7 to 14.7)
Risk difference (95% CI), percentage points	Reference	−0.4 (−2.6 to 2.5)	−1.7 (−5.7 to 3.4)	−3.6 (−7.6 to 3.5)
Trial 3				
Estimate, % (95% CI)	10.5 (8.9 to 12.4)	10.2 (7.9 to 13.2)	10.0 (7.6 to 13.3)	9.7 (7.5 to 12.8)
Risk difference (95% CI), percentage points	Reference	−0.3 (−1.9 to 1.7)	−0.6 (−2.5 to 2.2)	−0.8 (−2.7 to 1.9)
Trial 4				
Estimate, % (95% CI)	10.0 (8.0 to 12.3)	9.5 (7.2 to 12.9)	9.1 (6.7 to 12.4)	8.9 (6.6 to 12.2)
Risk difference (95% CI), percentage points	Reference	−0.5 (−1.9 to 1.6)	−0.8 (−2.5 to 1.3)	−1.1 (−2.7 to 1.1)
**Hospital: 12/y**				
Trial 2				
Estimate, % (95% CI)	6.8 (4.8 to 9.0)	6.5 (5.1 to 8.3)	5.7 (3.4 to 8.9)	4.5 (2.1 to 9.3)
Risk difference (95% CI), percentage points	Reference	−0.3 (−2.1 to 1.4)	−1.1 (−4.0 to 2.2)	−2.3 (−5.1 to 2.2)
Trial 3				
Estimate, % (95% CI)	7.8 (6.3 to 9.3)	7.5 (6.3 to 8.9)	7.4 (6.1 to 8.8)	7.2 (6.0 to 8.7)
Risk difference (95% CI), percentage points	Reference	−0.2 (−1.5 to 1.0)	−0.4 (−1.9 to 1.2)	−0.5 (−2.0 to 1.1)
Trial 4				
Estimate, % (95% CI)	7.9 (6.5 to 9.6)	7.5 (6.3 to 9.0)	7.3 (6.0 to 8.7)	7.1 (5.9 to 8.6)
Risk difference (95% CI), percentage points	Reference	−0.4 (−1.6 to 1.0)	−0.6 (−2.1 to 0.8)	−0.8 (−2.3 to 0.7)
**Hospital: 43/y**				
Trial 2				
Estimate, % (95% CI)	6.8 (3.2 to 10.4)	6.5 (3.1 to 10.4)	5.7 (3.1 to 8.3)	4.5 (2.2 to 7.2)
Risk difference (95% CI), percentage points	Reference	−0.3 (−1.9 to 1.5)	−1.1 (−4.7 to 1.6)	−2.3 (−6.0 to 1.4)
Trial 3				
Estimate, % (95% CI)	7.8 (6.4 to 9.4)	7.6 (6.2 to 9.2)	7.4 (6.3 to 8.7)	7.2 (6.2 to 8.5)
Risk difference (95% CI), percentage points	Reference	−0.2 (−1.5 to 1.0)	−0.4 (−2.0 to 1.1)	−0.6 (−2.2 to 1.0)
Trial 4				
Estimate, % (95% CI)	8.0 (6.6 to 9.7)	7.6 (6.4 to 9.3)	7.4 (6.4 to 8.6)	7.2 (6.3 to 8.4)
Risk difference (95% CI), percentage points	Reference	−0.4 (−1.6 to 1.0)	−0.6 (−2.4 to 0.8)	−0.8 (−2.5 to 0.7)

^a^ Surgeon and hospital volumes indicate the number of operations performed per year.

^b^ The reference value is lowest-volume surgeon within each hospital volume category.

**Figure.**
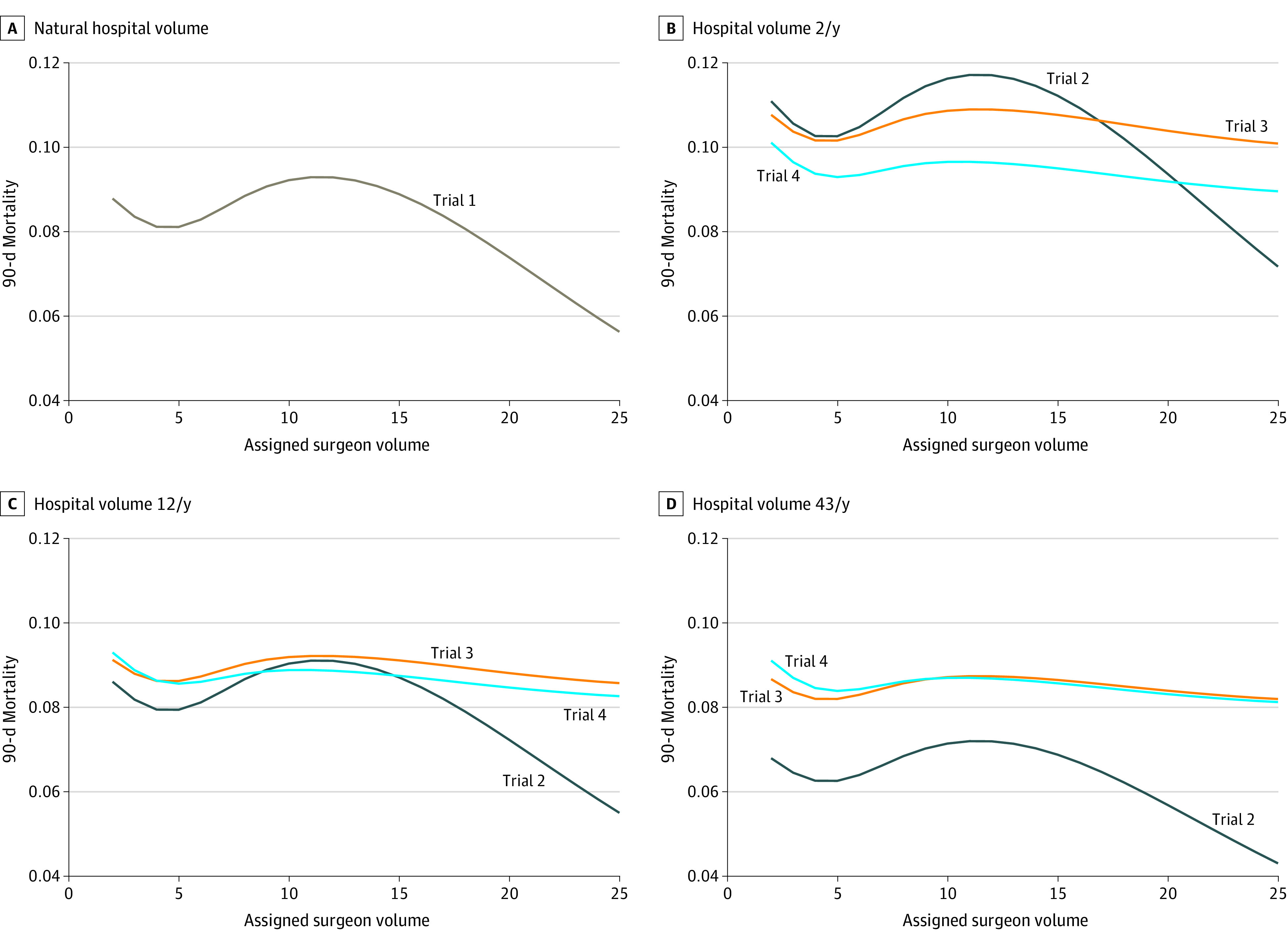
Estimates of 90-Day Postoperative Mortality for Each Target Trial The surgeon volumes displayed range from the 5th to 95th percentiles of observed surgeon volume.

The corresponding risk differences in 90-day postoperative mortality are presented in Table 3, relative to a surgeon who performed 2 pancreatectomies per year (for trial 1) or one who performed 2 pancreatectomies in the past year at a hospital that performed a lower (10th percentile, 2/y), median (12/y), or higher (90th percentile, 43/y) number of pancreatectomies in the past year (for trials 2-4). The CIs around the estimates were generally wide. Sensitivity analysis results are summarized in eFigures 4 and 5 in the Supplement.

For trial 1, the estimated risk difference in 90-day mortality was −2.6 percentage points (95% CI, −5.7 to 3.2 percentage points) for an operation by surgeons who performed 2 pancreatectomies (10th percentile) in the past year (without changing the distribution of available surgeons with these volumes) compared with an operation by surgeons who performed 25 pancreatectomies (95th percentile) in the past year. For trial 2, the corresponding risk difference estimate was −2.3 percentage points (95% CI, −5.1 to 2.2 percentage points) at median-volume hospitals and −2.3 percentage points (95% CI, −6.0 to 1.4 percentage points) at higher-volume hospitals. For trial 3, the corresponding risk difference estimate was −0.5 percentage points (95% CI, −2.0 to 1.1 percentage points) at middle-volume hospitals and −0.6 percentage points (95% CI, −2.2 to 1.0 percentage points) at higher-volume hospitals. For trial 4, the risk difference estimate was −0.8 percentage points (95% CI, −2.3 to 0.7 percentage points) at median-volume hospitals and −0.8 percentage points (95% CI, −2.5 to 0.7 percentage points) at higher-volume hospitals.

### Hospital Operative Volume

Mortality estimates stratified by surgeon operative volume rather than hospital operative volume are presented in eFigure 3 in the Supplement. For the emulation of trial 2, the risk differences between median- vs low-volume hospitals, high- vs low-volume hospitals, and high- vs median-volume hospitals among lower-volume surgeons were −4.1 (95% CI, −7.3 to −1.1) percentage points, −4.2 (95% CI, −8.2 to −0.2) percentage points, and 0.0 (95% CI, −4.0 to 3.9) percentage points, respectively; and among higher-volume surgeons, they were −2.9 (95% CI, −7.1 to −0.6) percentage points, −2.9 (95% CI, −9.4 to −0.1) percentage points, and 0.0 (95% CI, −4.2 to 2.2) percentage points, respectively. For the emulation of trial 3, the corresponding risk differences between median- vs low-volume hospitals, high- vs low-volume hospitals, and high- vs median-volume hospitals among lower-volume surgeons were −2.5 (95% CI, −5.3 to −0.1) percentage points, −2.5 (95% CI, −5.3 to −0.7) percentage points, and 0.0 (95% CI, −0.9 to 0.8) percentage points, respectively; and among higher-volume surgeons they were −2.9 (95% CI, −7.1 to −0.6) percentage points, −2.9 (95% CI, −9.4 to −0.1) percentage points, and 0.0 (95% CI, −4.2 to 2.2) percentage points , respectively. For the emulation of trial 4, the corresponding risk differences between median- vs low-volume hospitals, high- vs low-volume hospitals, and high- vs median-volume hospitals among lower-volume surgeons were −2.1 (95% CI, −4.7 to 0.3)percentage points, −1.9 (95% CI, −4.5 to 0.5) percentage points, and 0.1 (95% CI, −1.0 to 1.4) percentage points, respectively; and among higher-volume surgeons, they were −1.8 (95% CI, −4.6 to 0.2) percentage points, −1.7 (95% CI, −4.5 to 0.2) percentage points, and 0.0 (95% CI, −0.9 to 1.0) percentage points, respectively.

## Discussion

In this cohort study, we demonstrated how to specify and emulate trials for the association between mortality and different surgeon and hospital operative volumes, using the example of patients undergoing pancreatectomy. This approach may be used to interpret prior volume-outcome studies and to characterize potential pitfalls for future research.

Trial 1 is unrealistic because it considers interventions on surgeon volume only. Trial 2 is an improvement because it considers joint interventions on surgeon volume and hospital volume, but it is still unrealistic because it disregards travel time. Ignoring travel time may result in confounding bias that cannot be adjusted for because of the zero probability (nonpositivity) of traveling long distances to reach lower-volume surgeons. Despite the multiple shortcomings of trials 1 and 2, previous observational analyses^3,5,11^ can be viewed as attempts to emulate these designs, and hence the relevance of their estimates for decision-making is questionable. Moreover, most prior studies have been cross-sectional rather than longitudinal, which makes their estimates even harder to evaluate.

Trials 3 and 4 considered better-defined interventions and explicitly incorporated travel time. Trial 3 did not distinguish between the effect of the surgeon’s (or hospital’s) volume and the effect of confounders on the association between a particular surgeon and mortality (Figure). But this is inconsequential for a patient selecting a surgeon according to operative volume. Conversely, for policy makers who must consider the mechanism behind operative volume thresholds for credentialing privileges, these estimates may not be meaningful. Because trial 4 accounted for surgeon and hospital characteristics, these estimates are most relevant for design of a future policy on surgeon and hospital volume. These results may be most useful for decision makers concerned with volume-outcome mechanisms, as well as populations with different characteristics. Although trials 2 to 4 may not be logistically feasible because of the more than 1000 intervention arms, researchers studying a smaller range of operative volumes could use the same methodology without loss of generality.

### Limitations

This study has several limitations. First, residual confounding is always a concern when observational data are analyzed. We attempted to eliminate this bias through adjustment for multiple potential confounders. Second, we directly incorporated driving distance into the specified interventions to avoid losing generalizability; researchers interested only in a subpopulation of patients who live near higher-volume surgeons and hospitals might instead restrict their study to these eligible patients. Third, because operations performed for patients who were not Medicare beneficiaries were not included in the data, the measurement of operative volume may have underestimated the total (Medicare and non-Medicare) operative volume of a surgeon or hospital. However, exocrine pancreatic cancer is a disease of the elderly (incident diagnosis is highest among men aged 65-69 years and women aged 75-79 years)^10^ and there is no reason to suspect that surgeons would operate only on patients with or without Medicare. Therefore, approximate rank preservation of surgeons and hospitals by their volume between these data and a nationally representative sample of patients (not limited to Medicare beneficiaries) can be expected. Fourth, in our emulations, patients could be assigned to a strategy only by using the observed data at surgery. Thus, only trials in which all patients assigned to a surgeon ultimately undergo an operation were emulated. If a high proportion of patients were to become inoperable or die in the preoperative period, estimates would differ from those obtained in truly randomized trials (even in the absence of other biases). This proportion could not be calculated because, in the Medicare data, recording of the time of diagnosis was inconsistent. Fifth, these target trials and their emulations are intended to estimate the effect sizes of interventions on patients selecting their surgeons and hospitals according to operative volume. However, emulating these trials would not help to estimate the effect size of surgeons increasing or decreasing their number of operations over time. Emulation of such a trial requires consideration of time-varying confounding and is described elsewhere.^13^

## Conclusions

In summary, for volume-outcomes research, it is important to specify a well-defined intervention. Through this cohort study that emulated 4 hypothetical trial designs, we illustrated that the results and their interpretations differ according to the hypothetical target trial of interest. Introducing bias with analytic choices that deviate from the intended trial may lead to erroneous decision making.

## Notes

Supplement. **eAppendix.** Covariates **eFigure 1. **Acyclic Graph **eFigure 2.** Proportion of Individuals Within the Driving Time Thresholds by Surgeon and Hospital Volume **eFigure 3.** Estimates of 90-Day Postoperative Mortality for Each Target Trial (Dynamic Regimens With Threshold of 3 Hours’ Travel Time), Stratified by Surgeon Volume **eFigure 4.** Estimates of 90-Day Postoperative Mortality for Each Target Trial (Dynamic Regimens With Threshold of 3 Hours’ Travel Time) **eFigure 5.** Estimates of 90-Day Postoperative Mortality for Each Target Trial (Dynamic Regimens With Threshold of 6 Hours’ Travel Time) **eMethods.** Analysis of Target Trials

## References

[1] Luft HS, Bunker JP, Enthoven AC. Should operations be regionalized? the empirical relation between surgical volume and mortality. N Engl J Med. 1979;301(25):1364-1369. doi:10.1056/NEJM197912203012503 503167

[2] Bach PB, Cramer LD, Schrag D, Downey RJ, Gelfand SE, Begg CB. The influence of hospital volume on survival after resection for lung cancer. N Engl J Med. 2001;345(3):181-188. doi:10.1056/NEJM200107193450306 11463014

[3] Birkmeyer JD, Siewers AE, Finlayson EVA, . Hospital volume and surgical mortality in the United States. N Engl J Med. 2002;346(15):1128-1137. doi:10.1056/NEJMsa012337 11948273

[4] Reames BN, Ghaferi AA, Birkmeyer JD, Dimick JB. Hospital volume and operative mortality in the modern era. Ann Surg. 2014;260(2):244-251. doi:10.1097/SLA.0000000000000375 24368634 PMC4069246

[5] Birkmeyer JD, Stukel TA, Siewers AE, Goodney PP, Wennberg DE, Lucas FL. Surgeon volume and operative mortality in the United States. N Engl J Med. 2003;349(22):2117-2127. doi:10.1056/NEJMsa035205 14645640

[6] Leapfrog Group. Complex adult and pediatric surgery. Published October 6, 2019. Accessed October 6, 2019. https://www.leapfroggroup.org/ratings-reports/surgical-volume

[7] Leapfrog Group. Complex adult and pediatric surgery. Accessed January 31, 2022. https://ratings.leapfroggroup.org/measure/hospital/complex-adult-and-pediatric-surgery

[8] Modrall JG, Minter RM, Minhajuddin A, . The surgeon volume-outcome relationship: not yet ready for policy. Ann Surg. 2018;267(5):863-867. doi:10.1097/SLA.0000000000002334 28628561

[9] Wanis KN, Madenci AL, Dokus MK, . The meaning of confounding adjustment in the presence of multiple versions of treatment: an application to organ transplantation. Eur J Epidemiol. 2019;34(3):225-233. doi:10.1007/s10654-019-00484-8 30673924

[10] GBD 2017 Pancreatic Cancer Collaborators. The global, regional, and national burden of pancreatic cancer and its attributable risk factors in 195 countries and territories, 1990-2017: a systematic analysis for the Global Burden of Disease Study 2017. Lancet Gastroenterol Hepatol. 2019;4(12):934-947. doi:10.1016/S2468-1253(19)30347-4 31648972 PMC7026711

[11] Krautz C, Nimptsch U, Weber GF, Mansky T, Grützmann R. Effect of hospital volume on in-hospital morbidity and mortality following pancreatic surgery in Germany. Ann Surg. 2018;267(3):411-417. doi:10.1097/SLA.0000000000002248 28379871

[12] Operative volume and mortality: patient selection of surgeon and hospital. GitHub. Updated October 8, 2021. Accessed February 10, 2022. https://github.com/CausalInference/volume-patient

[13] Madenci AL, Wanis KN, Cooper Z, . Strengthening health services research using target trial emulation: an application to volume-outcomes studies. Am J Epidemiol. 2021;190(11):2453-2460. doi:10.1093/aje/kwab170 34089045 PMC8799904

