# Supplementary material for: Comparison of Mortality Risk With Different Surgeon and Hospital Operative Volumes Among Individuals Undergoing Pancreatectomy by Emulating Target Trials in US Medicare Beneficiaries
Source: JAMA Netw Open. 2022 Mar 10;5(3):e221766. doi: 10.1001/jamanetworkopen.2022.1766 (PMC8914572; doi:10.1001/jamanetworkopen.2022.1766)
Supplement: Supplement. — eAppendix. Covariates eFigure 1. Acyclic Graph eFigure 2. Proportion of Individuals Within the Driving Time Thresholds by Surgeon and Hospital Volume eFigure 3. Estimates of 90-Day Postoperative Mortality for Each Target Trial (Dynamic Regimens With Threshold of 3 Hours’ Travel Time), Stratified by Surgeon Volume eFigure 4. Estimates of 90-Day Postoperative Mortality for Each Target Trial (Dynamic Regimens With Threshold of 3 Hours’ Travel Time) eFigure 5. Estimates of 90-Day Postoperative Mortality for Each Target Trial (Dynamic Regimens With Threshold of 6 Hours’ Travel Time) eMethods. Analysis of Target Trials [file jamanetwopen-e221766-s001.pdf]

Supplemental Online Content

Madenci AL, Wanis KN, Cooper Z, et al. Comparison of mortality risk with different surgeon and hospital operative volumes among individuals undergoing pancreatectomy by emulating target trials in US Medicare beneficiaries. *JAMA Netw Open*. 2022;5(3):e221766. doi:10.1001/jamanetworkopen.2022.1766

eAppendix. Covariates

eFigure 1. Acyclic Graph

eFigure 2. Proportion of Individuals Within the Driving Time Thresholds by Surgeon and Hospital Volume

eFigure 3. Estimates of 90-Day Postoperative Mortality for Each Target Trial (Dynamic Regimens With Threshold of 3 Hours' Travel Time), Stratified by Surgeon Volume

eFigure 4. Estimates of 90-Day Postoperative Mortality for Each Target Trial (Dynamic Regimens With Threshold of 3 Hours' Travel Time)

eFigure 5. Estimates of 90-Day Postoperative Mortality for Each Target Trial (Dynamic Regimens With Threshold of 6 Hours' Travel Time)

eMethods. Analysis of Target Trials

This supplemental material has been provided by the authors to give readers additional information about their work.

© 2022 Madenci et al. *JAMA Network Open.*

eAppendix. Covariates

Operative information, specifically whether beneficiaries underwent pancreatectomy for the indication of pancreatic malignancy, was obtained from the Medicare Inpatient Claims file based on International Classification of Diseases diagnosis and procedure codes. The surgeon who performed each procedure was identified via a unique provider identification number designated by the "primary operator" field of the inpatient Medicare data.

Covariates used to emulate randomization included age, gender, race, pre-operative inpatient status, comorbidity history (including myocardial infarction, dementia, atrial fibrillation, chronic kidney injury, chronic obstructive pulmonary disease, congestive heart failure, and stroke or transient ischemic attack), and year of operation. We used data from the American Hospital Association Annual Survey of Hospitals to determine the characteristics of hospitals. History of relevant comorbidities was obtained the Medicare Master Beneficiary Summary File. Estimated travel time by driving was obtained using OpenStreetMap© through the interface osrm in R. Isochrone polygons containing all locations within 90 minutes were created around each included hospital based on ZIP code centroids. Patient ZIP code centroids were then queried to determine if the driving travel time to a hospital was within 90 minutes. For simplicity of modeling, we assume that all combinations of surgeon operative volume and hospital operative volume could exist, within covariate patterns. However, combinations of strategies involving the highest volume surgeons and the lowest volume hospitals should be interpreted with caution. For example, at low volume hospitals performing 2 operations per year, the highest volume surgeon performed 19 operations in the past year.

© 2022 Madenci et al. *JAMA Network Open.*

eFigure 1. Acyclic Graphs

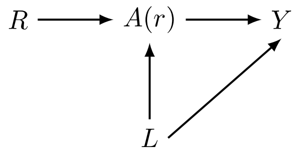

eFigure 1A. Causal directed acyclic graph for Hypothetical Trial #1 (assignment to surgeon volume only), in which the compound intervention R is randomly assigned and precedes the relevant version of surgeon. Node R denotes a patient's assignment to a surgeon with a specific operative volume, $A(r)$ denotes a patient's selection of a particular surgeon with the specified operative volume r, L denotes covariates associated with both the particular surgeon selected and mortality, and Y represents mortality at the end of follow-up.

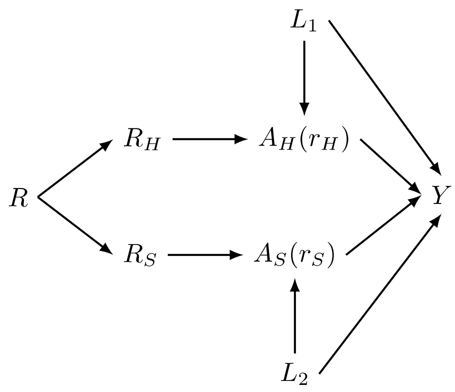

eFigure 1B. Causal directed acyclic graph for Hypothetical Trial #2 (assignment to surgeon volume and hospital volume), in which the compound intervention R denotes a random joint assignment of surgeon volume and hospital volume; R_H denotes the hospital volume component of R; R_S denotes the surgeon volume component of R; $A_H(r_H)$ denotes a patient's selection of a particular hospital with the specified operative volume r_H, $A_S(r_S)$ denotes an patient's selection of a particular surgeon with the specified operative volume r_S, L_1 denotes covariates associated with both the particular hospital selected and mortality, L_2 denotes covariates associated with both the particular surgeon selected and mortality, and Y denotes mortality at the end of follow-up.

This supplemental material has been provided by the authors to give readers additional information about their work.

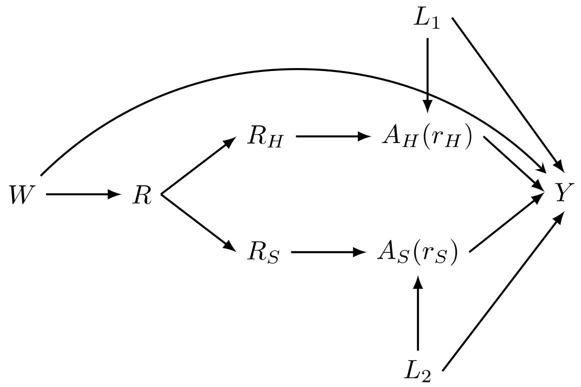

eFigure 1C. Causal directed acyclic graph for Hypothetical Trial #3 (dynamic assignment to surgeon volume and hospital volume, accounting for travel distance), in which the compound intervention R denotes a random joint assignment of surgeon volume and hospital volume; R_H denotes the hospital volume component of R; R_S denotes the surgeon volume component of R; $A_H(r_H)$ denotes a patient's selection of a particular hospital with the specified operative volume r_H, $A_S(r_S)$ denotes a patient's selection of a particular surgeon with the specified operative volume r_S, W denotes travel time from the nearest surgeon with volume ≥ R_S and hospital with volume ≥ R_H; L_1 denotes covariates associated with both the particular hospital selected and mortality, L_2 denotes covariates associated with both the particular surgeon selected and mortality, and Y denotes mortality at the end of follow-up.

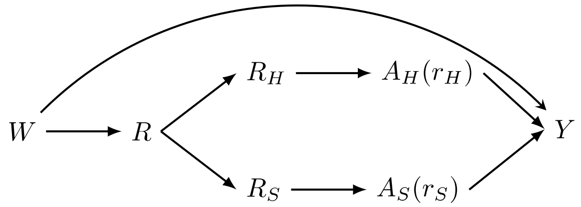

eFigure 1D. Causal directed acyclic graph for Hypothetical Trial #4 (dynamic assignment to surgeon volume and hospital volume, accounting for travel distance and multiple treatment versions), in which the compound intervention R denotes a random joint assignment of surgeon volume and hospital volume; R_H denotes the hospital volume component of R; R_S denotes the surgeon volume component of R; $A_H(r_H)$ denotes a patient's selection of a particular hospital with the specified operative volume r_H, $A_S(r_S)$ denotes an patient's selection of a particular surgeon with the specified operative volume r_S, W denotes travel time from the nearest surgeon with volume ≥ R_S and hospital with volume ≥ R_H; and Y denotes mortality at the end of follow-up.

This supplemental material has been provided by the authors to give readers additional information about their work.

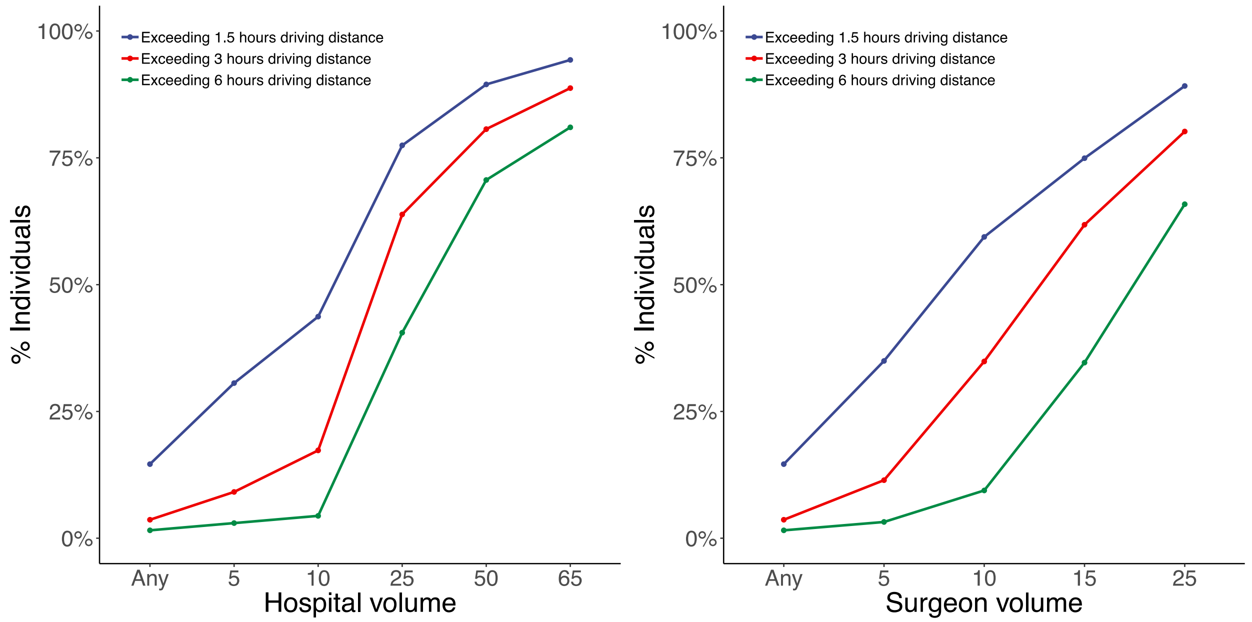

eFigure 2. Proportion of Individuals Within the Driving Time Thresholds by Surgeon and Hospital Volume

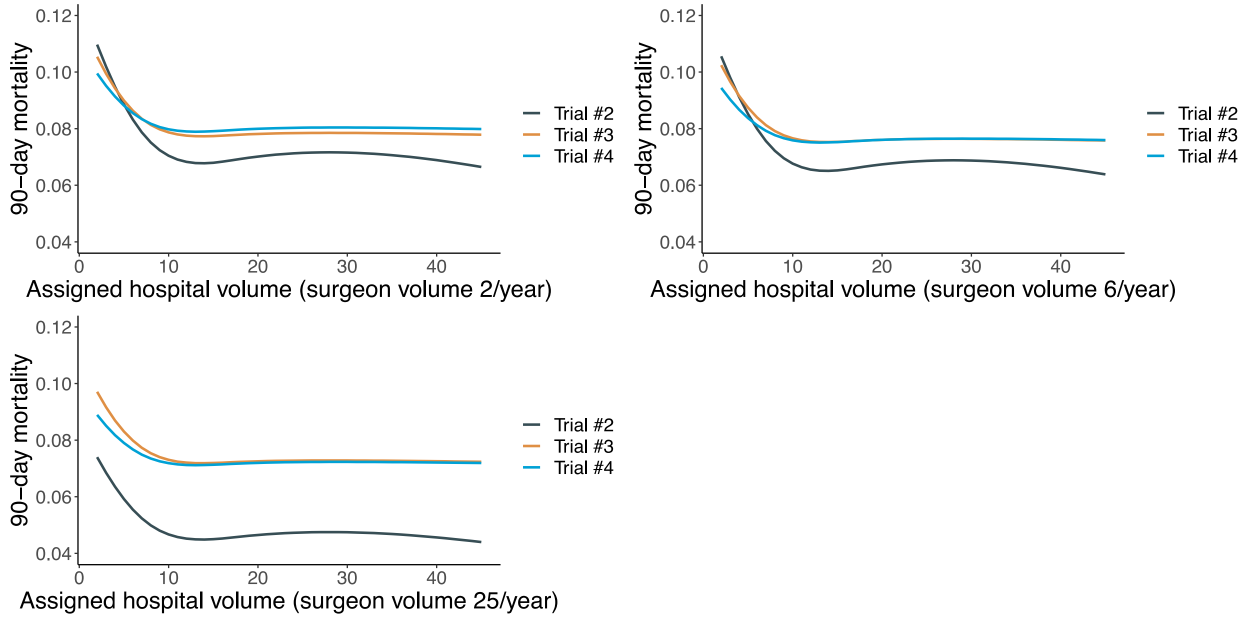

eFigure 3. Estimates of 90-Day Postoperative Mortality for Each Target Trial (Dynamic Regimens With Threshold of 3 Hours' Travel Time), Stratified by Surgeon Volume

This supplemental material has been provided by the authors to give readers additional information about their work.

© 2022 Madenci et al. *JAMA Network Open.*

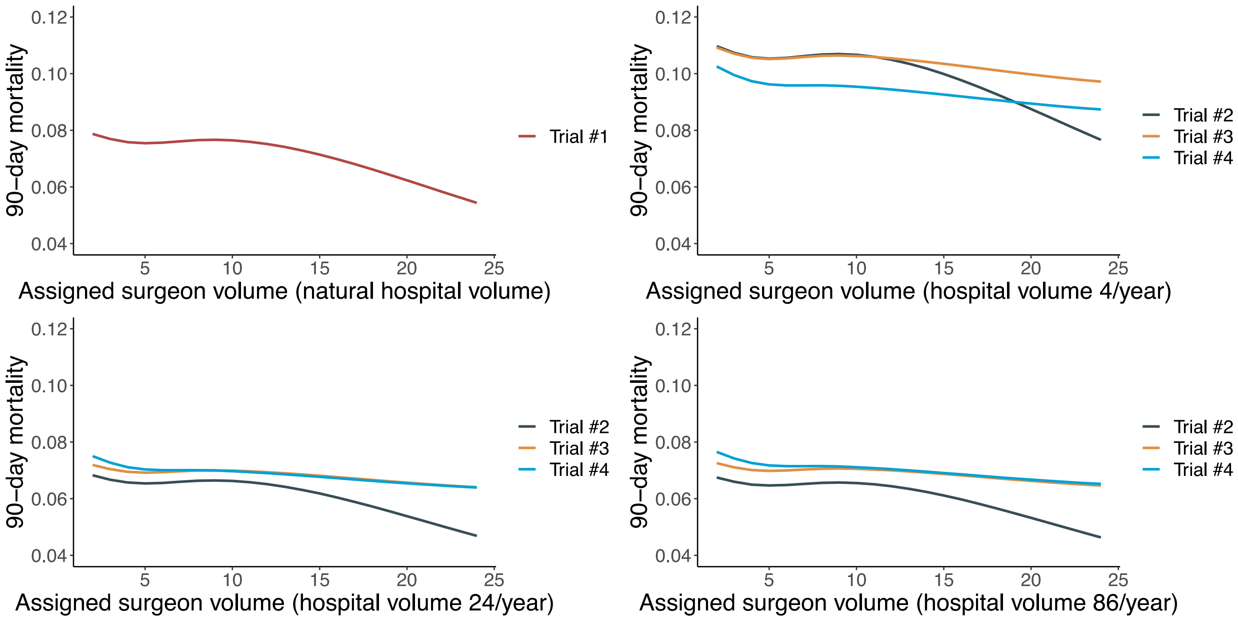

eFigure 4. Estimates of 90-Day Postoperative Mortality for Each Target Trial (Dynamic Regimens With Threshold of 3 Hours' Travel Time)
(The surgeon volumes displayed range from the 5th to 95th percentiles of observed surgeon volume.)

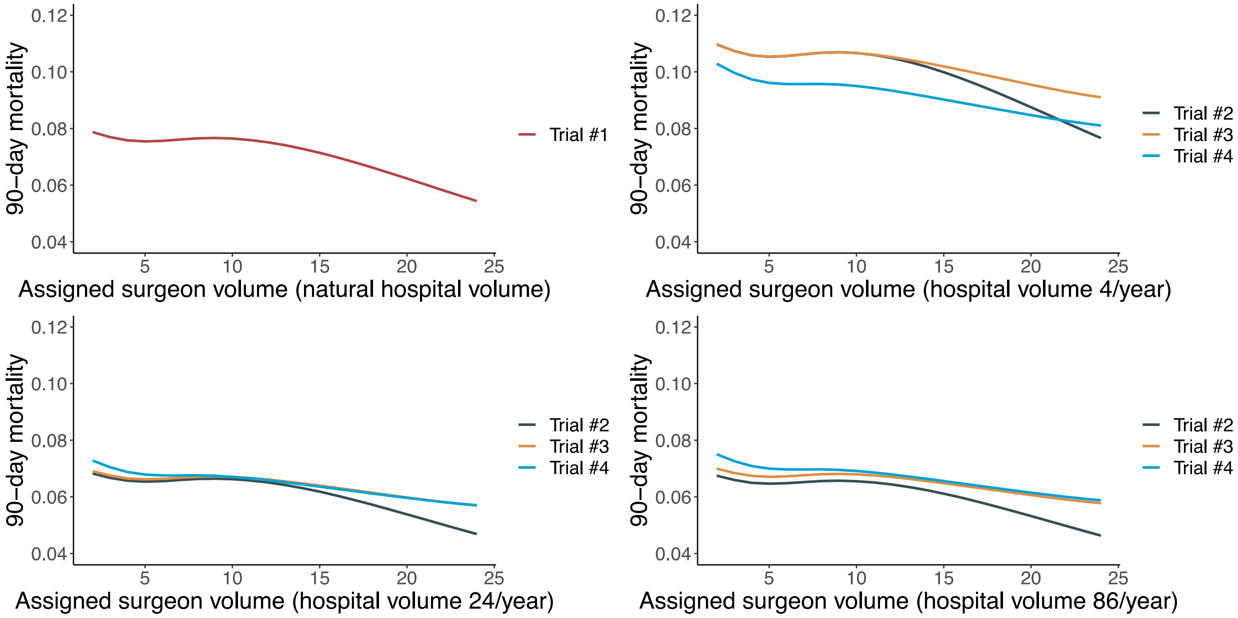

eFigure 5. Estimates of 90-Day Postoperative Mortality for Each Target Trial (Dynamic Regimens With Threshold of 6 Hours' Travel Time)
(The surgeon volumes displayed range from the 5th to 95th percentiles of observed surgeon volume.)

This supplemental material has been provided by the authors to give readers additional information about their work.

© 2022 Madenci et al. *JAMA Network Open.*

eMethods. Analysis of Target Trials

1.3.1 Analysis of Target Trials #1-4

Under full adherence to randomization, the intention to treat and per protocol effects are equivalent and could be estimated under each intervention arm non-parametrically or by fitting the following logistic model:

$$\hat{\mathbb{E}}\Big[Y\big|R_S = r_S, R_H = r_H)\Big] = \text{expit}\Big[\alpha_0 + \alpha_1^T f(r_S) + \alpha_2^T f(r_H) + \alpha_3 r_S r_H\Big] \quad (1)$$

Y is an indicator for 90-day mortality, R_S denotes surgeon volume (for all trials), R_H denotes hospital volume (for trials #2-4), and $f(\cdot)$ denotes a flexible functional form using restricted cubic splines with 4 knots.

1.3.2 Emulation of Analysis of Target Trials #1 and #2

The regression model used to emulate the analysis of the target trials is similar to Equation (1), but with the addition of baseline covariates to emulate randomization:

$$\sum_{l \in L}\Big[\mathbb{E}\Big(Y\big|R_S = r_S, R_H = r_H, L_0 = l_0\Big)p(L_0 = l_0)\Big] \quad (2)$$

$$= \mathbb{E}_{l \in L}\mathbb{E}\Big[Y\big|R_S = r_S, R_H = r_H, L_0 = l_0)\Big] \quad (3)$$

One way to estimate the conditional mean in Equation (3) is by fitting the following logistic model:

$$\hat{\mathbb{E}}\Big[Y\big|R_S = r_S, R_H = r_H, L_0 = l_0)\Big] = \quad (4)$$

$$\text{expit}\Big[\alpha_0 + \alpha_1^T f(r_S) + \alpha_2^T f(r_H) + \alpha_3 r_S r_H + \alpha_4^T l_0\Big]$$

We could then compute Equation (3) by computing the population sample average of each covariate pattern.

1.3.3 Emulation of Analysis of Target Trial #3

Given the dynamic regime, the Equation (4) above must be modified:

$$\hat{\mathbb{E}}\Big[Y = 0\big|R_S = g(r_S), R_H = g(r_H), L_0 = l_0)\Big] \quad (5)$$

where, for each individual, $g(r)$ takes the value of r, if the individual is within 1.5 hours of a surgeon with operative volume r; the value of the maximum volume surgeon within 1.5 hours of the individual, if the individual is >1.5 hours from a surgeon with the specified operative volume; or the observed surgeon operative volume, if the individual is not within 1.5 hours of *any* surgeon. The analogous rule is applied for hospitals.

1.3.4 Emulation of Analysis of Target Trial #4

$$\hat{\mathbb{E}}\Big[Y = 0\big|R_S = g(r_S), R_H = g(r_H), L_0 = l_0, L_1 = l_1)\Big] \quad (6)$$

For surgeons, such covariates in L_1 included age, gender, and working at more than one hospital; for hospitals, such covariates in L_1 included availability of a cardiac intensive care unit, hospital type (for profit, not for profit, or government non-Federal), teaching category (major, minor, or non-teaching), and size (1-99 beds, 100-399 beds, or $\geq$400 beds).

This supplemental material has been provided by the authors to give readers additional information about their work.

© 2022 Madenci et al. *JAMA Network Open.*
